# Impact of Oral Diseases and Conditions on Oral Health-Related Quality of Life: A Narrative Review of Studies Conducted in the Kingdom of Saudi Arabia

**DOI:** 10.7759/cureus.18358

**Published:** 2021-09-28

**Authors:** Mushir Mulla

**Affiliations:** 1 Department of Oral and Dental Health, College of Applied Health Sciences, Qassim University, Ar Rass, SAU

**Keywords:** saudi arabia, oral health related quality of life, quality of life, oral health, health

## Abstract

Oral health-related quality of life (OHRQoL) is a novel concept that has evolved over the past two decades. The World Health Organization (WHO) has also recognized it as a significant part of the Global Oral Health Program (2003). Information on OHRQoL gives better understanding about feelings and perceptions on an individual level. It also helps us to understand the impact of oral health on the lives on the patients and their family. It is now well documented that oral diseases and conditions impact people’s life. Some of the oral diseases/conditions like caries, dental fluorosis, tooth loss, periodontal disease, dental injuries, oral cancer, dental anomalies, craniofacial disorders, and many more have got negative impact on QoL. This paper identifies the various literatures published on the impact of oral diseases and conditions on OHRQoL in the population of Saudi Arabia. Although numerous researches can be found in other countries, the data on Saudi Arabian population are limited, leading to the need for carrying out more research in this area.

## Introduction and background

It is now well documented that oral diseases and conditions impact people’s life [1]. Oral diseases have functional, emotional, and social consequences and oral health-related pain can disrupt people’s food choices and speech, thereby diminishing the quality of life (QoL) [2,3]. Some of the oral diseases and conditions like caries, dental fluorosis, tooth loss, periodontal disease, dental injuries, oral cancer, dental anomalies, craniofacial disorders, and many more have got negative impact on QoL. Lately, various authors have demonstrated the relationship between these oral diseases/conditions with QoL in various populations.

In 1984, the concept of health was revised by the World Health Organization (WHO) and it is defined as "the extent to which an individual or group is able to realize aspirations and satisfy needs, and to change or cope with the environment. Health is a resource for everyday life, not the objective of living; it is a positive concept, emphasizing social and personal resources, as well as physical capacities" [4]. Thus, the concept of health is wide and it depends on various elements such as socioeconomic status, religious beliefs, cultural values, and individual perception.

Oral health-related quality of life (OHRQoL) is a novel concept that has evolved over the past two decades. OHRQoL is “a multidimensional construct that reflects (among other things) people's comfort when eating, sleeping, and engaging in social interaction; their self-esteem; and their satisfaction with respect to their oral health” [5]. It is also related with psychological factors, social factors, functional factors, and experience of pain or discomfort [6]. Various oral conditions have been reported in the literature as having an impact on OHRQoL. However, there is a shortage of data concerning the OHRQoL in the population of Saudi Arabia. Therefore, this review was conducted to identify the literature on the oral health status and OHRQoL in Saudi Arabia.

## Review

Various databases such as PubMed, Scopus, Web of science, Google scholar, and EBSCO were used for literature search and all the papers studying the relationship between oral diseases/conditions and QoL among people in Saudi Arabia were collected. Studies conducted in English were included. The search criteria included various oral diseases such as dental caries, dental fluorosis, tooth loss, periodontal disease, dental injuries, oral cancer, and dental anomalies. Studies evaluating the OHRQoL through assessment of functional, psychological, and social factors and experience of pain/discomfort in relation to oral diseases by means of validated tool were selected.

Tooth loss and edentulism affect dietary choice, masticatory function, and nutritional level. Study was conducted that measured the impact of tooth loss on the OHRQoL in adult patients pursuing dental care. Arabic version of 14‑item questionnaire of Oral Health Impact Profile (OHIP‑14 Ar) was used for the assessment. The authors presented that the severity of impact on OHRQoL increases with the higher number of teeth loss, thereby leading to higher oral impairment. Study participants with more than 10 teeth loss showed the highest OHIP‑14 score indicating greater oral impairment [7].

OHRQoL in patients with both maxillary and mandibular complete denture and also those with either the maxillary or the mandibular complete denture have been compared. The OHIP-EDENT (Oral Health Impact Profile in Edentulous Adults) questionnaire was used to asses 55 patients. It was found that the patients with complete dentures in both the jaws were less satisfied than the patients with single complete denture [8].

A cross-sectional study measuring OHRQoL among elderly individuals with edentulous jaws aged 65 years was reported. Arabic version of the Geriatric Oral Health Assessment Index (GOHAI-Ar) was used for the assessment of OHRQoL. Results of the study showed that an overall GOHAI-Ar score was 27.68 ± 0.54 on a scale of 0-60. The authors stated that this lower GOHAI-Ar score of 27.68 ± 0.54, which is suggestive of poor OHRQoL, may be directly related to their underlying malnutrition, diabetes, and any other medical condition that has direct influence on the OHRQoL [9]. 

Missing teeth are routinely replaced with fixed dental prostheses. It was found that dental plaque and gingival inflammation inevitably occur when proper oral hygiene measures were not applied [10,11]. To improve patients’ OHRQoL and to ensure the durability of the prosthodontic appliances, postoperative oral hygiene instructions and patient awareness should be increased [12].

Over the years, implants have become the treatment of choice for replacing missing tooth [13,14]. Researchers have studied the effect of immediate and delayed implant loading into the extracted site on the QoL in pre- and post-insertion period. QoL was better among the patients where dental implants were loaded immediately as compared to the delayed loading of dental implant [15].

There are several studies that reported that the dental caries have negative impacts on OHRQoL in populations of different ages [16-18]. The effect of dental caries experience and untreated dental decay and on OHRQoL was examined in working adults. The OHRQoL was evaluated using the OHIP-14 questionnaire. Significant caries (SiC) and decayed missed filled surfaces (DMFS) indices were used as dental health indicators. The results indicated that the SiC score could statistically significantly predict the OHIP score [17].

Another condition that affects QoL is malocclusion. Children were assessed to identify the relationship between malocclusion severity, as assessed by the Dental Aesthetic Index, and children’s QoL, as assessed by the Arabic version of Child Perception Questionnaire for 11- to 14-year-old children (CPQ11-14). These findings suggest that only very severe malocclusion had an impact on the QoL of the participants [19]. Authors have reported positive relationship between malocclusion and poor OHRQoL among older adults after assessing five occlusal traits such as overjet, overbite, crossbite, missing teeth, and displacement of contact point [20]. Another study carried out in Jeddah stated that vertical discrepancy in occlusion has got a negative impact on OHRQoL in both the genders. The negative impact is emphasized on the psychological disability, physical pain, psychological discomfort, and functional limitation domains [21].

Severe fluorosis can also have a negative effect on smile esthetics and produce functional problems, affecting self-confidence, causing discomfort, and probably disturbing social roles from a young age [22]. No reports were documented stating the effect of fluorosis on OHRQoL among children in Saudi Arabia.

Oral problems are one of the consequences of inadequate control of diabetes. The oral manifestations of diabetes consist of bacterial, viral, and fungal infections, delayed wound healing, increase in incidence and severity of dental caries, gingivitis, periodontal diseases, and burning mouth syndrome [23]. Based on the results of the survey conducted in Saudi Arabia, it seems that OHRQoL is adversely affected among the subjects having type 1 diabetes mellitus as compared to non-diabetic subjects [24].

The supporting tissues of the teeth are typically affected by periodontitis and this could potentially produce the impacts on individual's OHRQoL [25]. Study carried out among 25 individuals in Saudi Arabia found that the impact of periodontitis on the patient’s QoL was statistically significant in two domains, namely psychological disability (P value 0.001) and physical pain (P value 0.004). The severity of periodontitis, in this current study, did not indicate a negative impact on functional limitation [26].

In addition, OHRQoL has also been found to be affected by dental trauma injuries, cleft lip and palate, dental fluorosis, and temporomandibular dysfunction [27-30]. But to the best of author’s knowledge, no literature was found on Saudi Arabian population.

Table 1 provides an overview of all the studies included in this literature review about the impact of oral diseases and conditions on OHRQoL in people of the Kingdom of Saudi Arabia. Of the total studies reviewed, three evaluated tooth decay, three malocclusions, one periodontal disease, two edentulism, one tooth loss, one fixed dental prosthesis, and one dental implant loading (immediate and delayed). Four studies were performed on adolescents (two on malocclusion and two on dental caries) and eight were on adults. No studies were performed on children. Five studies were conducted in Riyadh region, three were conducted in Jeddah, and one each were conducted in Hafar Al-Batin, Mecca, Al-Khobar, and Al baha. It is important to mention that of the 13 provinces that make up the Kingdom of Saudi Arabia, the studies were carried out only in four provinces. This indicates that there is significant research gap about OHRQoL in Saudi Arabia. The OHRQoL instruments used in these studies were OHIP-14, OHIP-EDENT, GOHAI, QoL-OS/DS, CPQ11-14, COHIP-19, and CHILD-OIDP. Although various instruments were used to measure OHRQoL in different oral diseases and conditions, all the studies reported a negative impact of oral diseases and conditions on OHRQoL.

**Table 1:** Characteristics of the studies included PI: Plaque Index; GI: Gingival Index; OHIP: Oral Health Impact Profile; OHIP-EDENT: Oral Health Impact Profile in Edentulous; GOHAI: Geriatric Oral Health Assessment Index; GOHAI-Ar: Geriatric Oral Health Assessment Index-Arabic; QoL-OS/DS: Quality of Life-Oral and Dental Subscale; DMFS: Decayed Missed Filled Surfaces; SiC: Significant Caries; DAI: Dental Esthetic Index; CPQ11-14: Child Perception Questionnaire for 11- to 14-year-Old Children; DMFT: Decayed Missed Filled Teeth; COHIP-19: Child Oral Health Impact Profile; Child-OIDP: Children's Version of the Oral Impacts on Daily Performance; IOTN: Index of Orthodontic Treatment Need.

S. no	Author/Year	Region	Study design	Sample size	Age group	Oral disease/condition (Instrument)	OHRQoL (Instrument)
1	Anbarserri NM et al., 2020 [7]	Riyadh	Cross-sectional	152	18 years and above	Tooth loss (PI, GI, and complete periodontal examination)	OHIP‐14
2	AlBaker AM, 2013 [8]	Riyadh	Cross-sectional	55	42-75 years	Single or both arches complete edentulism (complete oral examination)	OHIP-EDENT and GOHAI
3	Alshammari M et al., 2018 [9]	Hafar Al-batin	Cross-sectional	200	65 years and above	Partial edentulism, single arch complete edentulism or both arches complete edentulism (complete oral examination)	GOHAI-Ar
4	Alrumyyan A et al., 2020 [12]	Riyadh	Cross-sectional	528	18 years and above	Fixed dental prosthesis (questionnaire)	OHIP‐14
5	Bahammam MA and Fareed WM, 2019 [15]	Jeddah	Experimental study	24	18-60 years	Dental implants (clinical examination)	QoL-OS/DS
6	Bukhari OM, 2020 [17]	Makkah	Cross-sectional	160	18-60 years	Dental (DMFS) and (SiC)	OHIP-14
7	Dawoodbhoy I et al., 2013 [19]	Al-Khobar	Cross-sectional	278	11-14	Malocclusion (DAI)	CPQ_11–14_
8	Agou S, 2020 [20]	Jeddah	Cross-sectional	87	18 years and above	Malocclusion (IOTN)	OHIP-14
9	Altouki et al., 2020 [21]	Jeddah	Cross-sectional	109	12-25	Malocclusion (IOTN)	OHIP-14
10	AlMutairi FFJ et al., 2020 [24]	Riyadh	Cross-sectional	40	12-15	Dental caries (DMFT) and gingival status (GI)	COHIP-19
11	AlZahrani et al., 2019 [2]	Al Baha	Cross-sectional	349	12-15	Dental caries (DMFT), gingival status (GI), and plaque scores (PI)	Child-OIDP
12	AlKattan R et al., 2017 [26]	Riyadh	Cross-sectional	25	18 years and above	Periodontal status (complete oral examination)	OHIP‐14

## Conclusions

There is always a need for greater number of studies to be documented regarding the OHRQoL. Information on OHRQoL gives better understanding about feelings and perceptions on an individual level, thereby helping to increase the chance of effective communication between health professionals and patients. It also helps us to understand the impact of oral health on the lives of the patients and their family. 

Although oral health is related closely to individual’s general health and QoL, very few literatures are available on Saudi Arabian population. Dental health professionals and public health programs should target their efforts not only toward prevention of dental/oral pain but also to improve OHRQoL. Thus, OHRQoL should also be measured after the dental treatment is provided so as to ensure that the treatment outcome has not only improved the oral health status but also the overall well-being of the patient. 
